# Idiopathic Granulomatous Mastitis

**DOI:** 10.1155/2024/6693720

**Published:** 2024-01-25

**Authors:** Christina Dilaveri, Amy Degnim, Christine Lee, Daniel DeSimone, Dan Moldoveanu, Karthik Ghosh

**Affiliations:** ^1^Mayo Clinic, Department of Medicine, Division of General Internal Medicine, Rochester, USA; ^2^Mayo Clinic, Department of Surgery, Division of Breast and Melanoma Surgical Oncology, Rochester, USA; ^3^Mayo Clinic, Department of Radiology, Division of Breast Imaging and Intervention, Rochester, USA; ^4^Mayo Clinic, Department of Medicine, Division of Infectious Diseases, Rochester, USA

## Abstract

Idiopathic granulomatous mastitis (IGM) is a rare, benign inflammatory disorder of the breast that is often underrecognized. The exact etiology and pathophysiology are unknown, but milk stasis is felt to play a role. Classically, this condition is noninfectious, but many cases are noted to be associated with *Corynebacterium* species. Most patients affected are parous women with a mean age of 35, and many have breastfed within five years of diagnosis. Patients typically present with a painful mass and symptoms of inflammation, and these features can sometimes mimic breast cancer. Biopsy is needed to make a definitive diagnosis, and noncaseating granulomas are found on core biopsy. Many patients have a waxing and waning course over a period of six months to two years. Goal of treatment is to avoid surgery given poor wound healing, high risk of recurrence, and poor cosmetic outcomes. Medical treatment is preferred and includes observation, antibiotics, steroids, and immune modulators such as methotrexate. In more recent years, topical and intralesional steroids have become the treatment of choice, with similar outcomes to oral steroids.

## 1. Introduction

Idiopathic granulomatous mastitis (IGM) is a benign, rare, chronic inflammatory disorder of the breast which was first described by Kessler and Wolloch [1] in 1972 and further detailed in a five-case series by Cohen in 1977 [2]. Also called nonpuerperal mastitis or granulomatous lobular mastitis, it has an annual prevalence of 2.4 in 100,000 and an incidence rate of 0.37% [3]. Its rarity presents both a diagnostic and therapeutic challenge to clinicians. Although the etiology remains unknown, it is thought to involve a local granulomatous inflammatory response to damaged ductal epithelium. The initiating process may include extravasation of intraluminal secretions, while some evidence suggests a possible infectious etiology [4]. IGM usually presents as an inflammatory process but can also be clinically and radiologically like breast cancer, requiring a biopsy to rule out malignancy, with the diagnosis often being one of exclusion. While significant progress has been made in recent years, definitive treatment remains controversial, with little consensus on the optimal treatment strategy. Conservative medical management is currently endorsed as first-line treatment, while surgery is generally reserved for refractory cases due to its significant morbidity and substantial recurrence rates. The aim of this paper is to review all facets of the diagnosis of IGM, including the demographics of patients affected by IGM, the proposed pathophysiology and etiologies, the clinical presentation and imaging features, histopathology, differential diagnosis, and the current treatment options.

## 2. Demographics

IGM has a prevalence of 2.4 in 100,000 [3]; however, this rate may be underestimated given underdiagnosis or misdiagnosis [5]. The majority of cases are unilateral, though there have been case reports of bilateral involvement [6, 7]. The mean age at presentation ranges from 32 to 35 years (8), but cases as young as 11 and as old as 83 have been reported [6, 8, 9]. There are also reports of the disease occurring in men and one report of IGM in a male-to-female transgender individual [10, 11]. No association has been reported between IGM and future breast cancer risk [8].

There is a strong relationship between IGM and a history of lactation and pregnancy. More than half of patients report had a pregnancy in the five years (range of 6 months to 6 years) preceding diagnosis [6, 12]. The mean breastfeeding duration was 3–36 months, and IGM onset occurred 6 months to 5 years after breastfeeding cessation [6, 8, 9]. IGM during pregnancy or breastfeeding is uncommon but has been reported [13]. There are rare cases of IGM in nulliparous women, and some of these are associated with elevated prolactin levels, either due to medications or pituitary tumors [6, 9].

IGM has been reported in individuals of all races without a well-established predisposition to one specific ethnic group. However, some studies report an association with nonwhite ethnicities, including those of Asian, Hispanic, and Middle Eastern descent [6]. In the continental United States, Hispanic ancestry is associated with IGM [14, 15].

## 3. Pathophysiology

Although the exact cause is unknown, several mechanisms have been proposed for the pathogenesis of IGM. Milk stasis is felt to play a crucial function and may explain the strong correlation between IGM, pregnancy, and breastfeeding. The process starts with intraluminal secretion, which in the setting of hormonal imbalances in estrogen-to-progesterone ratios or hyperprolactinemia can result in milk stasis. Milk protein may trigger an autoimmune process within the interstitial breast tissue related to microtrauma from breastfeeding, stasis, or other causes [4]. This process could potentially be modulated by specific bacteria such as *Corynebacterium* species [4, 9]. Damage to the epithelial lining of the ducts causes ductal contents to efflux from the lumen of the duct into the adjoining lobular connective tissue. A local inflammatory response develops in response to this ductal efflux. Lymphocytes and macrophages migrate to the periductal zones and noncaseating granulomas form [4]. Microscopically, IGM is defined by noncaseating granulomas concentrated in the lobules. These contain Langerhans giant cells, plasma cells, lymphocytes, and epithelioid histiocytes. Organized microabscesses within a neutrophilic infiltrate are seen with loss of acinar structure and damaged ducts [4]. Larger abscesses can result from a confluence of multiple microabscesses [9, 16]. Gram-positive Coryneform bacteria may also be present in association with the abscesses.

Cystic neutrophilic granulomatous mastitis (CNGM) is a rare subtype of IGM linked to *Corynebacterium* species infection [17–23]. It is recently recognized as a cause of granulomatous breast inflammation and has been linked with specific species of lipophilic *Corynebacterium* species, including *Corynebacterium kroppenstedtii* and *Corynebacterium tuberculostearicum* [20, 22, 23]. These bacteria can survive in the suppurative lipogranulomas and are often inaccessible to water-soluble beta-lactam antibiotics. These Gram-positive bacilli are identified in only half of CNGM cases as they are difficult to grow using standard culture techniques. Using specific media and longer incubation periods can increase the yield of these cultures [17].

## 4. Proposed Etiologies

While the exact etiology of IGM is unknown, several hypotheses have been proposed. Granulomatous mastitis can be divided into two groups: specific and nonspecific. The former has an identifiable cause, such as an infectious or systemic autoimmune process. The latter has no identifiable cause and is usually subdivided into IGM and CNGM.

Several precipitating factors have been proposed for IGM, including infection, alpha-1 antitrypsin deficiency, oral contraceptive use, lactation, pregnancy, hyperprolactinemia, smoking, trauma, diabetes, and autoimmune disease [6, 8, 9, 12]. However, pregnancy, lactation, and hyperprolactinemia are the only conditions clearly associated with IGM.

Laboratory analysis of IGM classically shows no positive cultures, yet growing evidence suggests a role for *Corynebacterium* species in this disease process, whether or not the diagnosis is specifically CNGM. The endogenous bacteria flora of the breast is similar to that of the rest of the body. However, the anatomy of the breast allows bacteria to access the ductal system through the nipple. Corynebacteria are part of normal skin flora and are unlikely pathogens under normal circumstances. However, *Corynebacterium* does cause mastitis in livestock [17], and when present in deeper tissues, it may drive an inflammatory response. *Corynebacterium* involvement in IGM is difficult to quantify accurately due to its fastidious nature and frequent classification as “normal flora” by microbiology labs. When present in cultures, it is often unclear whether these organisms represent colonization, contamination, or infection [20]. Detecting purulent matter in an abscess or greater than 10^4^colony-forming units/mL dominant *Corynebacterium* species is considered diagnostic of infection [17]. However, it is conceivable that even smaller concentrations could drive chronic inflammation if present in normally sterile tissue. Modern genomic sequencing techniques could be helpful in advancing understanding of the role of *Corynebacterium* in IGM. Four *Corynebacterium* species have been detected in IGM cases, especially CNGM. *Corynebacterium kroppenstedtii* is the most common isolate and differs from the other species by its lipophilic nature and positive esculin test [17, 18, 20, 22].

Alpha-1 antitrypsin deficiency has been proposed as a possible cause of IGM. Alpha-1 antitrypsin is a glycoprotein produced in the liver and is a member of the serine protease inhibitor family. Its primary function is to prevent the harmful effects of secreted proteases from activated neutrophils, including proteinase 3, elastin, and cathepsin G [9]. Deficiency in alpha-A antitrypsin leads primarily to liver and lung pathologies. It is an autosomal dominant condition most prevalent in Caucasians of European or North American descent [16]. In some cases, it is associated with clinical symptoms of panniculitis, which histologically can show predominantly lobular inflammation, similar to that seen in IGM. A case report from 2001 [24] describes a 37-year-old female with alpha-1 antitrypsin deficiency diagnosed with IGM. In the absence of other causal factors, it was suggested that the deficiency could represent an etiological factor.

Oral contraceptives (OCPs) have been considered a potential etiological factor as they can increase breast luminal secretions [9]. Several case series have identified a relationship between OCP use and IGM [25–27]. However, several other case series have failed to reproduce these findings, bringing the relationship under doubt [28–30]. The strength of the association between OCP use and IGM ranges between 0% and 42% in the literature [4].

In 1994, Rowe [31] described a case of IGM in a patient with prolactinoma. Given the association between hyperprolactinemia and galactorrhea, this condition has been proposed as a possible contributor to the etiology of IGM [32–34]. However, many studies evaluating IGM do not provide prolactin levels, while others show no relationship between IGM and prolactin levels; however, hyperprolactinemia is thought to be responsible for at least a fraction of IGM cases [9, 29].

Other causal associations of IGM have also been investigated. While other types of mastitis, such as periductal mastitis, are associated with tobacco use [35, 36], the relationship between IGM and smoking is less clear [4, 9]. Because IGM often responds to steroids and other immunomodulator medications, an autoimmune etiology has been suggested, but no definitive autoimmune pathway or mechanism has been established, and no specific serological markers are associated with IGM. Cases with a comorbid autoimmune disorder constitute only a minor fraction of all cases [9, 16]. Classic serological tests used for other autoimmune disorders, such as ANA and rheumatoid factor, are often negative and noncontributory in establishing a diagnosis of IGM [16].

## 5. Clinical Presentation

The most common clinical presentation of IGM is a unilateral, tender palpable mass within the breast [6]. It can also present as multiple simultaneous areas of peripheral, rarely central, masses or inflammation. Up to 50% of patients develop erythema and swelling. Approximately 37% present with an abscess [4, 6]. The lesions may occur in any quadrant of the breast, with the subareolar region being the least affected. Bilateral breast masses are rare, with very few case reports noting bilateral involvement [7]. Skin erythema, edema, induration, and peau d'orange changes can also be seen. If the nipple is involved, retraction, ulceration, and discharge have been documented. Axillary lymphadenopathy can be present, raising the suspicion of breast cancer. In cases of IGM, axillary lymphadenopathy is often reactive and may occur in 28% of patients [6]. When an abscess is present, a sinus or fistula formation may develop as a result of disease progression or as a complication of a prior percutaneous biopsy or aspiration [6]. The disease tends to develop over weeks to months and often mimics malignancy. Symptoms can persist from six months to two years, but overall, the disease tends to be self-limiting.

## 6. Imaging Features

While there are no pathognomonic imaging features for diagnosing IGM, imaging can provide supporting evidence for diagnosis and may help document the size, number, and location of lesions. Ultrasound and MRI can identify fluid collections or abscesses, and ultrasound can guide therapeutic aspirations as well as intralesional therapies. Serial imaging can help monitor lesions over time, evaluate treatment response, and detect new disease sites or recurrence early.

There are no specific mammographic features for IGM. In the literature, IGM most commonly presents as a unilateral focal or regional asymmetry [6, 37] with or without associated architectural distortion. Alternatively, it can present as one or more irregular masses. The area of IGM can also be associated with coarse heterogeneous calcifications. In some cases, IGM can be mammographically occult, likely due to the high breast density seen in the young, often lactating patients [37].

Since mammographic findings of IGM are generally nonspecific, breast cancer cannot easily be excluded. The fairly common finding of axillary lymphadenopathy and skin or trabecular thickening with peau de orange may sometimes raise suspicion for inflammatory breast cancer.

Targeted breast ultrasound is usually performed for further characterization. The sonographic appearance of IGM includes lobulated, heterogeneous, hypoechoic masses with indistinct, irregular, or angular margins [37]. Indistinct, hyperechoic rims, and internal vascularity are often seen. Tubular extensions are often described [8] and may represent involvement of the segmental ductal/lobular tree and perilobular inflammation. The masses often demonstrate posterior acoustic shadowing, which makes the distinction with breast cancer even more challenging. Fluid collections or abscesses may be present in 7 to 54.0% of cases [6]. Skin thickening, edema, obliteration of subcutaneous fat, and sinus tracts to the skin or between lesions can sometimes be identified. While biopsy is often still indicated to rule out a malignant process, multiple irregular, heterogeneous hypoechoic lesions in the context of a compatible clinical history may suggest IGM rather than breast cancer.

Breast MRI may sometimes be helpful to document the extent and progression of disease, especially when mammogram and ultrasound are inconclusive. Heterogeneously enhancing or rim-enhancing masses are the most common findings [37]. IGM cases are often noted to have mixed progressive and plateau enhancement kinetics. Most patients have a T2 hyperintense-enhancing mass with or without nonmass enhancement [6]. The margins and shapes of the masses can range from ill-defined to well-circumscribed, round, oval, or irregular. T2 hyperintense, peripherally enhancing, and centrally nonenhancing masses are characteristics of abscess formation. MRI can also characterize nipple retraction, sinus tracts, and parenchymal distortion. MRI cannot reliably distinguish breast cancer from granulomatous mastitis; a biopsy is usually required.

## 7. Histopathology

A biopsy is the gold standard for IGM diagnosis. Since many of the clinical and radiographic features of IGM overlap with those of breast cancer, a biopsy is performed to confirm the diagnosis. Core needle biopsy is the gold standard with a sensitivity of 96%, while fine-needle aspiration has a sensitivity of only 21.1% [6]. Biopsy findings typically show granulomatous lesions centered on the breast lobule [16]. In addition to evaluating tissue with Gram stain and culture/sensitivity, stains for acid-fast bacilli and fungi should be obtained to rule out breast tuberculosis or sarcoidosis, which can both result in granuloma formation.

On histopathology, nonnecrotizing granulomas are present in combination with a localized infiltrate of multinucleated giant cells, epithelioid histiocytes, lymphocytes, and plasma cells [16]. Granulomas are lobulocentric, and lymphocytes, plasma cells, and polymorphic leukocytes are consistent with a chronic inflammatory picture. Organized sterile microabscesses can occur with neutrophilic infiltrates. Inflammation that extends into adjacent lobules can indicate a higher severity [4]. The damaged ducts show loss of acinar structures in the involved breast parenchyma [16]. A typical feature is the absence of microorganisms as IGM is by definition noninfectious; however, staining for bacteria, including Gram stain, acid-fast staining, and fungal staining, PAS and GMS are recommended to help rule out infection-associated granulomas. The absence of caseating necrosis helps rule out tuberculosis. Also, distinguishing between granulomas affecting lobules versus the ducts helps with this differential [4, 16].

In cases of CNGM, suppurative lipogranulomas are comprised of a central lipid space encircled by neutrophils, which can, in turn, be surrounded by epithelioid histiocytes microscopically [20, 22]. In cases of CNGM, *Corynebacterium* species are often isolated on culture and can sometimes be seen on Gram stain.

## 8. Differential Diagnosis

IGM does need to be differentiated from other autoimmune and granulomatous conditions (Table 1). Bacterial infectious causes of granulomatous inflammation include tuberculosis, leprosy, and cat-scratch disease. Fungal disorders that can cause a granulomatous reaction include histoplasmosis, cryptococcosis, and coccidiomycosis [6, 8]. Actinomycosis can cause chronic draining breast abscesses and may mimic IGM. Foreign body reactions to silicone and beryllium, as well as fat necrosis can also have a similar clinical presentation [6]. Autoimmune causes of granulomatous inflammation include Crohn's disease, sarcoidosis, and several vasculitides (granulomatosis with polyangiitis, giant cell arteritis, Takayasu's arteritis, and Churg–Strauss syndrome) [6, 9]. IgG4-RD (related disease) mastitis is on the differential and is diagnosed exclusively on histopathology as a dense lymphoplasmacytic infiltrate with storiform fibrosis and obliterative phlebitis [38]. Inflammatory breast cancer must be ruled out.

The workup of the patient includes a focused history and physical examination (Table 2). Historical features such as fevers, night sweats, cough, occupational exposure, or other systemic symptoms can point toward other diagnoses. Chest X-ray can rule out mediastinal lymphadenopathy or pulmonary infiltrates, which may suggest tuberculous mastitis or sarcoidosis [39]. Gram stain, acid-fast Ziehl–Neelsen staining, fungal staining, PAS, and GMS staining are recommended on tissue samples. Mycobacterial and fungal cultures at the time of aspiration or biopsy, as well as bacterial cultures, help rule out other infectious etiologies [7]. Fungal serologies can be performed if there is high suspicion for this etiology, especially in endemic areas. In addition to history and exam, autoimmune serologies can help rule out autoimmune conditions [6].

## 9. Treatment

There is no consensus on the optimal treatment for IGM. A multidisciplinary evaluation and treatment should be pursued with consideration of various modalities, including observation, antibiotics, corticosteroids, or other immunosuppressants. Surgery can be offered in select patients who do not respond to conservative therapy.

Overall, IGM has a self-limited (albeit often protracted) course and may resolve spontaneously within approximately two years, with some reports describing spontaneous improvement in as little as 6–12 months [4]. However, given the significant symptom burden and impact on quality of life, a therapeutic approach that can optimize symptom control with minimal adverse outcomes is desirable. Below, we review the available therapeutic strategies and provide a rational approach to treatment sequencing based on the clinical features of the disease.

## 10. Observation

Approximately 50% of cases of IGM resolve spontaneously without any specific intervention [4, 14]. For this reason, observation with close follow-up is a reasonable first-line therapy for limited and mild cases. It is important to note that observation is only appropriate after a diagnosis has been made, which typically involves diagnostic imaging, aspiration of any fluid collections, and tissue diagnosis with biopsy. This approach would be more suitable for small, focal lesions with mild symptoms. Several studies have noted spontaneous resolution within 6–12 months [4]. In these situations, supportive therapy such as pain control with oral anti-inflammatory medications and reassurance may be appropriate.

## 11. Medical

### 11.1. Antibiotics

As many patients first present with breast erythema, induration, or fluid collections, most are started on empiric antibiotic therapy for presumed bacterial mastitis. If an abscess is present, it is critical to obtain cultures prior to antibiotic initiation, as bacterial culture may be falsely negative with prior antibiotic therapy. The choice of antibiotic should be tailored to culture results, and treatment should be stopped in the absence of growth.

Given the preponderance of *Corynebacterium* species in CNGM, antibiotic coverage with amoxicillin–clavulanate or doxycycline should be strongly considered as an empiric initial therapy [40, 41]. In such cases, antibiotic courses of at least two weeks may be required [17, 18, 23]. When abscesses are present, aspiration for culture and susceptibility testing is recommended prior to initiating treatment.

## 12. Steroids

Medical therapy is the first-line treatment for moderate and severe IGM. It can provide effective symptomatic control and often results in durable resolution of the inflammatory lesions. Once the diagnosis is definitively established with biopsy, negative cultures, and lack of improvement after antimicrobial therapy, the mainstay of medical treatment for IGM consists of steroid therapy, an approach first proposed in 1980 [42]. Side effects of systemic corticosteroids include mood changes, elevated blood pressure, glucose intolerance, peptic ulcer disease, and Cushing syndrome. At high doses and when taken for a prolonged period of time, patients can also develop opportunistic infections, myopathies, neuropsychiatric symptoms, and osteoporosis. The goal of steroid therapy is to use the minimal effective dose for the shortest duration. However, if tapering is premature, there is a risk of rebound. Infectious causes must be appropriately treated prior to starting steroids.

### 12.1. Oral Steroids

Earlier studies of oral corticosteroids suggested starting with doses of 60 mg of prednisone daily (49). Current evidence suggests beginning with lower doses, ranging from 30 to 40 mg daily. Initially, shorter courses of four weeks were prescribed, but it is now recognized that courses of 3–6 months are optimal to prevent recurrence [43]. Recent research indicates that topical steroid creams or intralesional steroids are effective and preferred (see below), but if oral steroids are utilized, starting at 30–40 mg of prednisone daily to obtain initial disease control, with an early rapid taper to 20 mg daily over a period of two to four weeks is recommended, depending on the response. This is followed by a more gradual taper over 3–6 months until complete discontinuation to prevent rebound symptoms.

### 12.2. Topical Steroids

In a study comparing topical, systemic, and combined topical and systemic steroids, authors compared prednisolone 0.125% applied to the affected breast twice daily on weekdays with breaks during the weekends compared with oral methylprednisolone 60 mg and 4 mg tapered slowly. Clinical and radiological responses were compared between therapies. Systemic side effects were significantly lower in the topical therapy group than in the groups that used systemic steroids. Complete clinical response was observed in 83.3% of patients and was similar in all three groups [44].

In another study evaluating the effect of topical steroids on IGM, prednisolone 0.125% pomade was applied to the breast twice daily on alternating days for four days with an interval of three days off, repeating on a weekly cycle, for an average of 8.2 weeks. The topical therapy achieved complete symptom control in all patients, including resolution of fistulae, skin erosions, and breast parenchymal lesions. Side effects were minimal in the topical steroid group. Over 37.2 months of follow-up, only 10.7% of patients relapse, which was successfully treated with a second course of topical steroids. Another study using a similar regimen, prednisolone 0.125% with a similar treatment regimen and duration, reported similar efficacy and recurrence rates and no side effects or steroid-related complications [45].

The skin of the breast is thin, and thus, medium-potency topical steroids should be used, such as prednisolone 0.125% or triamcinolone 0.1%. Preparations include pomades, creams, and ointments. Barriers can be applied over the treated skin to ensure undecided opted contact with the topical preparation. Based on the study results above, the preferred approach is twice daily topical steroid application on alternating days, for a total of four days a week and a duration of eight weeks.

### 12.3. Intralesional Steroids

Intralesional steroid therapy for IGM has been studied in several small case series [46–49]. For instance, triamcinolone acetonide 40 mg/mL intralesional injections combined with topical steroids compared to systemic steroids and combined local therapy were as effective as systemic steroids with significantly fewer side effects [46]. Response to systemic therapy was faster, with a mean recovery of 11.7 weeks compared to 20.2 weeks. A second study compared intralesional injection of 80–160 mg of triamcinolone acetonide (40 mg/mL) with observation [47]. Time to symptom resolution was significantly shorter in patients treated with intralesional corticosteroids, with a median time of 2 months, versus 11.5 months in the observation group. All patients undergoing steroid injection had resolution of symptoms.

In a study comparing intralesional steroid administration versus systemic steroids, the authors reported no difference in clinical regression, response to treatment, side effects, and recurrence rates. Only minor side effects were reported in either group [48]. Because of this, intralesional steroid administration should also be considered as a potential treatment method in lieu of systemic steroids.

A study of IGM in pregnant women evaluated intralesional and topical steroid administration. Methylprednisolone 40 mg was injected intralesionally, and prednisolone 0.125% pomade was applied twice daily on alternate days for four weeks. Patients with diffuse disease or with lesions in more than two quadrants received two-site injections, 5 cm apart. All patients in the study achieved a complete response, requiring at most two courses of treatment. Corticosteroids side effects were not observed in any of the patients [49].

Based on these studies, intralesional corticosteroids injection appears to be an effective and safe alternative, demonstrating comparable results to oral steroids and minimal adverse effects.

## 13. Nonsteroidal Immunosuppressants

### 13.1. Methotrexate

In patients with refractory disease, who do not respond to steroids or are unable to tolerate a taper, methotrexate has been studied as a steroid-sparing agent. Methotrexate is a folic acid analogue and binds to dihydrofolate reductase to decrease cell proliferation and thymidylate and purine synthesis. It suppresses lymphocyte proliferation and adenosine accumulation and exerts an anti-inflammatory effect by inhibiting other folate-dependent enzymes [3]. Methotrexate is used as a steroid-sparing agent for other granulomatous conditions such as sarcoidosis and giant cell arteritis (36). As it is teratogenic, it is not recommended for women who are planning to have children, who are pregnant, or who are lactating. Doses of 10–15 mg weekly for a duration of 3–6 months with a slow taper have been studied in patients with IGM [4]. Historically, symptom resolution rates are approximately 83%, with recurrence rates of 17% [39, 50], similar to the rates seen with oral steroids.

In a recent publication, authors evaluated methotrexate as initial therapy for patients with IGM. A total of 21 patients were included, and those with no contraindications to methotrexate were treated with methotrexate monotherapy, adding low doses of prednisone in cases of severe inflammation. All patients showed initial complete remission, and 17.6% experienced relapse after tapering that did respond well to low-dose methotrexate re-treatment [50]. One patient experienced continued hair loss; the remaining drug reactions were mild. Overall, this study demonstrates that methotrexate is well tolerated and can be used as first-line therapy in select cases.

### 13.2. Azathioprine

Azathioprine is another immunosuppressant studied as a steroid-sparing agent. It works by suppressing formation of antibodies and affects both cellular and humoral immunity by reducing numbers of T, B, and natural killer cells. In addition, it inhibits prostaglandin synthesis and has been used as an antirejection medication and for treatment of other autoimmune diseases [4]. Few studies have evaluated azathioprine in IGM. It remains an option for patients who cannot tolerate steroid discontinuation and have contraindications to using methotrexate.

### 13.3. Mycophenolate Mofetil

In a single case report of a patient with IGM who had failed both systemic corticosteroids and methotrexate, mycophenolate mofetil 1500 mg twice daily and intralesional triamcinolone resulted in overall resolution of symptoms after 4–6 months [51].

## 14. Other Medical Therapies

### 14.1. Bromocriptine

Because hyperprolactinemia is a possible etiological factor in developing IGM by overstimulating breast parenchymal tissue, medications that lower prolactin levels have been proposed as potential therapeutic agents. In a case series, 16 patients were treated with a combination of steroids and bromocriptine 5–10 mg daily. A favorable response was seen in 31% of patients [4], suggesting that in patients with IGM, adding bromocriptine to steroids may be beneficial.

## 15. Surgery

Prior to steroids being studied in 1980, IGM was managed with surgical excision [4]. Surgical approaches have included wide local excision, partial mastectomy, mastectomy, and percutaneous drainage with or without fistula tract removal. The primary focus of surgery for IGM has been to remove the affected inflamed tissue with a margin of surrounding normal breast tissue. In patients with extensive disease, this could result in mastectomy because of multiple lesions, diffuse involvement, or history of sinus tract involvement. Recurrence rates range from 5.5 to 50%, with more recent studies citing rates at the lower end of the spectrum, possibly because early interventions consisted of limited drainage rash recent surgical approaches excise of all diseased and necrotic tissues [39].

Breast surgery for IGM is associated with poorer cosmetic outcomes, partly because surgery is now reserved for medically refractory cases with more extensive and progressive disease. If the entire affected area is not removed and simple drainage is performed, recurrence rates are high. Conversely, if all involve tissue is removed, wound healing is slow and missing tissue can result in breast deformity. With surgery, excision of all diseased tissue is the key to complete healing, and the incision is usually packed to allow healing by secondary intention [4]. Once the wound is clean and healing, delayed closure procedures can be considered. Depending on the volume of breast tissue lost relative to the overall breast size, plastic surgery involvement can be critical for achieving adequate cosmesis. Due to the contaminated field, implants are not utilized, but delayed fat-grafting and autologous flaps are good options to restore the lost breast volume [52]. As IGM is a benign disease, mastectomy is reserved for truly extensive disease that cannot be managed with a breast-conserving approach.

Given the risks of recurrence, scarring, and poor cosmesis, the primary indications for surgery include lack of response to medical therapy, progression despite adequate therapy, and persistent fistulous/sinus tract disease. Since IGM usually has a self-resolving course, the overall goal of therapy is to provide symptomatic relief, reassure, support, and closely follow patients through the period of time until the disease process has run its course. However, in those cases that do not respond to medical therapy, especially with persistent fistulous tracts, surgical treatment can be successful when undertaken appropriately [53]. The American Society of Breast Surgeons has opened a registry of patients with IGM, which hopefully will expand outcomes data on the surgical management of this disease.

## 16. Conclusion

IGM is a rare inflammatory disorder of the breast; however, it may be underrecognized and may be more common than currently reported. Given its response to steroids and other immune-modulatory medications, it has a presumed autoimmune basis, although questions remain about a possible infectious etiology involving *Corynebacterium*. Workup to rule out other infectious or unusual systemic inflammatory etiologies is recommended. Given the significant clinical and radiographic overlap with breast cancer, a tissue biopsy is needed to make a histologic diagnosis. Up to 50% of cases will resolve spontaneously within two years, so initial conservative strategies are recommended, with careful selection of cases to treat based on symptoms and extent of disease. Initial strategies can include antibiotics, corticosteroids, and immune modulatory medications. Antibiotic choice should ideally be targeted towards *Corynebacterium* species given the preponderance of this bacterium in IGM cases. Topical and intralesional steroids demonstrate equivalent efficacy with minimal side effects compared to oral steroids; these topical and intralesional approaches are preferred initial treatment options for patients with IGM, especially those with limited disease. When oral steroids are needed, they should be started at higher doses and slowly tapered over time to avoid rebound inflammation. Surgery is generally reserved for cases that are refractory and progressive in spite of medical therapy; surgery is often successful but usually requires a prolonged healing period and delayed reconstructive procedures.

## Figures and Tables

**Table 1:** Differential diagnosis of IGM.

IGM
(i) Cystic neutrophilic granulomatous mastitis
Inflammatory breast cancer
Infectious mastitis
(i) Bacterial
* Corynebacterium* species, especially *C. kroppenstedtii* and *C. tuberculostearium*
Periductal mastitis
Actinomyces abscess
(ii) Tuberculous mastitis
(iii) Leprosy
(iv) Cat scratch disease
(v) Fungal
Histoplasmosis
Cryptococcosis
Coccidiomycosis
(vi) Protozoal
Schistosomiasis
Diabetic mastopathy
Autoimmune
(i) Vasculitis
(ii) Wegener's granulomatosis
(iii) Giant cell arteritis
(iv) Takayasu's arteritis
(v) Churg–Strauss syndrome
(vi) Breast sarcoidosis
(vii) Crohn's disease
Foreign body granulomas
(i) Silicone, paraffin, PAAG injections, and beryllium
IgG4-RD mastitis

**Table 2:** Workup of granulomatous breast disease.

Diagnosis	Tests to consider
Sarcoidosis	ACE level, chest radiograph, and serum calcium
Tuberculosis	PPD, MTB QuantiFERON Gold, chest radiograph, acid fast tissue stain, and mycobacterial tissue culture
Infectious mastitis	Tissue culture for bacteria, fungi, and atypical mycobacteria; Gram stain, fungal stain, acid fast stain; fungal serologies if fungal infection suspected in endemic areas
Wegener's granulomatosis	ANCA antibody, PR3 antibody, CRP, creatinine, UA, and chest radiograph
Giant cell arteritis	ESR/CRP
Takayasu's arteritis	ESR/CRP, CBC (may show anemia or elevated platelets), CT chest angiography with contrast, or CT abdomen angiography with contrast
Churg–Strauss syndrome	CBC with differential to evaluate for eosinophilia, histopathology of affected organ, and electromyelogram to diagnosis peripheral neuropathy or mononeuritis multiplex
Crohn's disease	Colonoscopy with ileal exam, small bowel imaging with CT or MR enterography, evidence of extraintestinal signs (erythema nodosum, pyoderma gangrenosum, episcleritis, scleritis, anterior uveitis or iritis, and primary sclerosing cholangitis)
IgG4-RD mastitis	Histopathology demonstrating classic findings; serum IgG4 levels not needed for diagnosis but can be supportive of diagnosis
Diabetic mastopathy	Characteristic histopathology in a patient with type I diabetes
Breast cancer	Histopathology, ideally from core needle biopsy
